# Evaluation of the relationship between dermatoglyphics and mandibular third molar impaction: A cross-sectional study

**DOI:** 10.12688/f1000research.123398.1

**Published:** 2022-09-29

**Authors:** Ashish Kapoor, Premalatha Shetty, Sameep S. Shetty, Srikant N., Nancy Aggarwal, Yash Merchant, Seyed Mohammed Riahi

**Affiliations:** 1 Department of Oral and Maxillofacial Surgery, Manipal College of Dental Sciences, Mangalore, Manipal Academy of Higher Education, a constituent of MAHE, Manipal, Karnataka, 575001, India; 2 Department of Oral Pathology and Microbiology, Manipal College of Dental Sciences, Manipal Academy of Higher Education, a constituent of MAHE, Manipal, Karnataka, 575001, India; 3 Department of Oral and Maxillofacial Surgery, Ratna Memorial Hospital, Pune, Maharashtra, 411016, India; 4 Department of Epidemiology, Birjand Institute of Medical Sciences, Birjand, 9717853577, Iran

**Keywords:** Dermatoglyphics, impacted tooth, third molar, fingerprint, young adult

## Abstract

**Background:** Dermatoglyphics can be utilised in clinical settings to identify those who are more likely to have impacted teeth. Additionally, dermatoglyphics looks to have potential as a non-invasive diagnostic method for predicting the presence or absence of an impacted tooth. The goal of this study was to look at the most common dermatoglyphic pattern in people who had or didn't have an impacted mandibular third molar teeth and see if there was a dermatoglyphic signature.

**Methods: **A cross-sectional study with 180 participants was conducted (90 cases and 90 controls). The rolling impression technique was used to apply blue duplicating ink to participants' fingertips, which was then recorded. There was an increase in the frequency of the whorl-plain pattern in the right-hand ring finger (60%; p=0.028) and left-hand little finger (33.3%; p=0.009), as well as the loop-ulnar pattern in the right-hand middle finger (74.4%; p=0.024) in individuals with a predisposition to the presence of impacted teeth.

**Results:** The left-hand little finger was found to be the most predictive for impaction in a forward stepwise binary logistic regression analysis.

**Conclusions:** Dermatoglyphics could be used as a non-invasive sign to predict whether or not a tooth is affected. Its value comes in early detection, which helps to avoid the surgical problems that come with symptomatic extraction of an impacted tooth.

## Introduction

Cumins and Midlo invented the term dermatoglyphics, which is taken from two Greek words – *derma* and *glyphics.* *Glyphe* means “carve” and *derma* means “skin” in English. ^1^ Dermatoglyphics is the study of the patterns of dermal ridges on the fingertips, palms, soles, and toes of the feet. During the sixth and seventh weeks of pregnancy, the baby develops thick pads called volar pads on the fingers, hands, and feet. Volar pads are derived from the mesenchyme and form on the extremities of the fingers, palms, and feet. After week 10 of pregnancy, the pads stop expanding, but the phalanges continue to extend, causing pattern orientation. Around the 13th week of pregnancy, ridge formation begins, and pattern formation is completed by the 19th week of pregnancy. ^2^

Finger, palm, and sole impressions are thought to be influenced by both the environment and heredity. It has been proven scientifically that no two people, including monozygotic twins, have the same fingerprints or other dermal ridge characteristics. As a result, fingerprints are unique to each person and do not change over time as a result of disease, ageing, or other factors. ^1^

Galton (1892) was one of the first to use distinct fingerprint ridgeline patterns or minutiae to investigate fingerprint individuality. Fingerprints are influenced by genetic and environmental variables. Dermatoglyphics are beneficial in a variety of ways, including assisting in the diagnosis of single-gene illnesses. The fact that each person’s fingerprint is unique adds to the argument that a person’s genetic features will be revealed by his or her fingerprint. ^3^

An impacted tooth is one that is unable to erupt into its normal functional position due to malposition, insufficient space, or other factors. A multitude of local and systemic factors that can act as roadblocks in an impacted tooth’s path to eruption prohibit it from erupting into its functional position within the projected chronological age. The interaction of local factors, such as the ectopic position of the tooth bud; ankylosis of the primary tooth; arch size-tooth size discrepancy; systemic factors, such as genetic, environmental, and endocrine disorders; and inherent genetic susceptibility, appears to be the driving force behind the impaction process. ^4^ There have been reports of racial and demographic differences in impacted teeth. Asian and Black populations had a lower prevalence rate of impaction, but those in Greece and Turkey had a greater prevalence rate. ^5^

The third molar is the last tooth to form and erupt in humans, as well as the tooth that is most likely to become impacted. Previous study has connected impacted third molars to pericoronitis, caries, periodontal issues, root resorption, and cysts or tumours of the jaw. The extraction of the impacted third molar has consequently become the most common treatment in the Department of Oral and Maxillofacial Surgery. ^6^ These changes are influenced by genes, developmental processes, and a refined diet. Nonetheless, a small number of studies have revealed a relationship between the mandible and a higher occurrence of impacted third molars. ^7^

Aside from radiographic diagnosis, an investigation strategy that is quick, cost-effective, and accurate is necessary. In this case, clinicians may profit from anticipating impaction and intervening at an early stage. There has been a paucity of research on predicting an individual’s likelihood of getting impaction in the field of dermatoglyphics. Teeth and fingerprints are both ectodermal. As a result, dermatoglyphics can be utilised as a marker to predict a variety of genetically susceptible disorders, bolstering the genetic basis.

This study’s purpose is to answer the following clinical question: is there a specific fingerprint pattern associated with impacted third molars? According to our hypothesis, there is a relationship between dermatoglyphics and mandibular tooth impaction. This study aimed to analyse the most common pattern of dermatoglyphics present in an individual with/without impacted mandibular third molar tooth and find the dermatoglyphic marker if any.

## Methods

### Study design and ethical issues

This comparative cross-sectional study was conducted in the city of Mangalore, in the district of Dakshin Kannada, in the southern Indian coastline region. The study was authorized by the Institutional Ethics Committee, Manipal College of Dental Sciences, Mangalore (Approval Date: 13.10.2018; Protocol Reference Number: 18061), and the subjects gave their written informed consent before participating.

### Participants, setting, and eligibility criteria

Individuals aged 20 years or older, residents of Karnataka and Kerala states, seeking dental care at Manipal College of Dental Sciences, Mangalore between 2018 and 2020 were eligible to participate. Those with physical limitations such as amputation of forelimbs or scars on their fingers, as well as those with impacted third molars with cystic lesions or neoplasms, were not allowed to participate.

### Sample size

Sample size: 180 (90 * 2)

the following formula was used to calculate the sample size:

$$n=\frac{\left(z\alpha \right)\surd 2p\;q+\mathrm{ZB}\surd (p1\;q1+p2\;q2){}^{2}}{{\left(p1-p2\right)}^{2}}$$

$$p=\frac{p1+p2}{2}$$

q = 100 − p

p1 = proportion of first group q1 = 100 − p1

p2 = proportion of second group q2 = 100 − p2

Zα = 1.96 at 95% confidence level ZB = 1.28 at 90% power

$$\begin{array}{c} n=90^{*}2\\ =180 \end{array}$$

With 95% confidence level and 90% power by assuming 47% will give whorl fingerprint pattern in first group and 24% will give in the second group ^8^ the sample size comes to be 90 for each group.

### Sampling procedure and data collection

Individuals with impacted third molars/partially erupted third molars/missing third molars with no history of extraction were included in the case group, which consisted of 90 subjects, whereas individuals with the full complement of erupted teeth (32 teeth) were included in the control group, which consisted of 90 subjects.

Non-probability quota sampling was utilized as the sample method. With blue duplicating ink, the ink process was employed to record the impression of fingerprints. The following supplies were used: a magnifying glass (×5), a cotton applicator, soap, water, tissue paper, and a study proforma sheet.

The hands of the participants were cleansed with soap and water, then dried with tissue paper to eliminate sweat, oil, and grime from the fingertips, ensuring a high-quality dermatoglyphic print. After that, a small dab of blue duplicating ink was put to the cotton applicator, and a thin coating of ink was smeared evenly on the fingertip. To record the impression on the research proforma sheet, the rolling impression technique was preferred. By rotating the finger from one side to the other (nail to nail), complete imprints were recorded. ^9^^,^^10^

Each digit was firmly pushed into the proforma sheet with constant and adequate pressure to achieve a high-quality impression. To minimize duplication, the blocks on the proforma sheet were numbered 1 to 5 for the right hand, beginning with the thumb and ending with the little finger, and 6 to 10 for the left hand, beginning with the thumb and ending with the little finger. A panoramic radiograph was taken for participants who had missing tooth/teeth without a history of surgery/extraction to confirm the presence of impacted tooth/teeth or congenital missing tooth/teeth.

### Study variables

Demographic variables were recorded. Impaction was used as a dependent variable, with all versions of fingerprints from all fingers being used as independent variables. All of the fingerprints were categorized into five different categories: loop-radial, loop-ulnar, whorl-plain, whorl-pocket, whorl-double loop, and arch-plain. Under the guidance of the forensic expert, the recorded prints were examined with a hand-magnifying glass. The patterns visualized were:

*Whorl pattern:* It has a concentric design in which the majority of the ridges make circuits around the core. They are further divided into: i) plain (concentric circles form the pattern around a core); ii) double loop (two loops form an S-shaped pattern); iii) accidental (irregular shaped pattern); and iv) central pocket loop (a loop with a whorl pattern at one end).

*Loop pattern* : It has ridges that are open and curve around only on the single extremity of the pattern and flow to the margin of the digit. They are further divided into: i) radial (loop ends towards the radial side); and ii) ulnar (loop ends towards the ulnar side).

*Arch pattern*: It has ridges passing from one margin of the digit to the other with a distally bowed sweep. They are further subdivided into: i) plain (the wave in the centre is blunt/low in height); and ii) tented (the wave in the centre is sharp/high in height). ^1^

### Statistical analysis

Statistical analysis was performed using IBM SPSS Statistics (RRID:SCR_016479) version 22.0 (IBM Inc., New Armonk, NJ, USA). The impaction with sex and the pattern of dermatoglyphics with each finger was compared using the chi-squared test. The prediction of impaction was analysed using forward stepwise binary regression analysis. Impaction was taken as the dependent variable, and the patterns of each finger of the individual were taken as the independent variables for prediction. The level of significance was set at p<0.05 in all analyses.

### Preprints

An earlier version of this article can be found on Research Square (doi: https://doi.org/10.21203/rs.3.rs-951125/v1 ).

## Results

**Figure 1.**
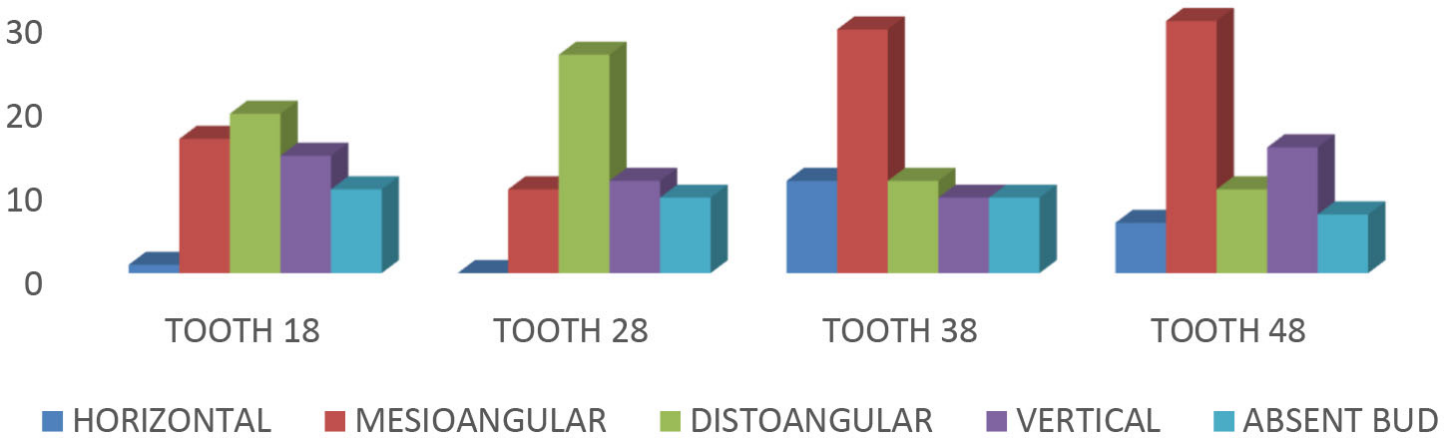
Type of impaction.

A total of 180 subjects were engaged in the study (90 cases and 90 controls). Female participants made up the majority (n=123; 68.3%) ( Table 1) (23). Mesioangular impaction was more common in mandibular teeth (38:42%; 48:44.1%), while distoangular impaction was more common in maxillary teeth (38:42%; 48:44.1%) (18:31.7%; 28:46.4%). The maxilla had missing tooth buds more frequently (18:16.7%; 28:16.1%) than the mandible (38:13%; 48:10.3%) ( Figure 1). A total of 42 of the 90 people (46.7%) with impacted teeth had all four teeth impacted, followed by 21 (23.3%) who had two impacted teeth ( Table 2).

**Table 1.** Association of variables with impaction.

Variable	Categories	N	Impaction		Chi-squared value	P-value
			No impaction, N (%)	Impacted third molar, N (%)		
Sex	Female	123	54 (60)	69 (76.7)	5.777	0.016 *
	Male	57	36 (40)	21 (23.3)		
Right-hand middle	Loop-R	2	2 (2.2)	0 (0)	16.169	0.024 *
	Loop-U	118	51 (56.7)	67 (74.4)		
	Whorl-plain	40	24 (26.7)	16 (17.8)		
	Whorl-pocket	1	1 (1.1)	0 (0)		
	Whorl-double loop	3	0 (0)	3 (3.3)		
	Whorl-accidental	1	0 (0)	1 (1.1)		
	Arch-plain	10	8 (8.9)	2 (2.2)		
	Arch-tented	5	4 (4.4)	1 (1.1)		
Right-hand ring	Loop-R	3	3 (3.3)	0 (0)	14.141	0.028 *
	Loop-U	72	41 (45.6)	31 (34.4)		
	Whorl-plain	94	40 (44.4)	54 (60)		
	Whorl-pocket	3	0 (0)	3 (3.3)		
	Whorl-double loop	1	0 (0)	1 (1.1)		
	Whorl-accidental	0	0 (0)	0 (0)		
	Arch-plain	6	5 (5.6)	1 (1.1)		
	Arch-tented	1	1 (1.1)	0 (0)		
Left-hand little	Loop-R	13	10 (11.1)	3 (3.3)	15.475	0.009 *
	Loop-U	110	55 (61.1)	55 (61.1)		
	Whorl-plain	46	16 (17.8)	30 (33.3)		
	Whorl-pocket	1	0 (0)	1 (1.1)		
	Whorl-double loop	1	1 (1.1)	0 (0)		
	Whorl-accidental	0	0 (0)	0 (0)		
	Arch-plain	9	8 (8.9)	1 (1.1)		
	Arch-tented	0	0 (0)	0 (0)		

* p<0.05. R, Radical; U, Ulnar.

**Table 2.** Distribution of the number of impacted teeth.

Number of teeth impacted	Count	%
1	19	21.10
2	21	23.30
3	8	8.90
4	42	46.70

The most common fingertip pattern amongst research participants was loop-ulnar, which was seen more than 50% of the time in eight fingers (right and left thumb, index, middle, and little fingers), followed by whorl-plain, which was seen more than 50% of the time in two fingers (ring finger of both right and left-hands). Less than 10% of the respondents reported seeing the other patterns ( Table 3).

**Table 3.** Finger wise distribution of the pattern.

Pattern	Right, N (%)					Left, N (%)				
	Thumb	Index	Middle	Ring	Little	Thumb	Index	Middle	Ring	Little
Loop-R	3 (1.7)	12 (6.7)	2 (1.1)	3 (1.7)	6 (3.3)	12 (6.7)	17 (9.4)	6 (3.3)	4 (2.2)	13 (7.2)
Loop-U	96 (53.3)	84 (46.7)	118 (65.6)	72 (40.0)	111 (61.7)	90 (50.0)	74 (41.1)	105 (58.3)	72 (40.0)	110 (61.1)
Whorl-plain	60 (33.3)	57 (31.7)	40 (22.2)	94 (52.2)	55 (30.6)	49 (27.2)	53 (29.4)	43 (23.9)	95 (52.8)	46 (25.6)
Whorl-pocket	1 (0.6)	3 (1.7)	1 (0.6)	3 (1.7)	1 (0.6)	1 (0.6)	0 (0.0)	3 (1.7)	2 (1.1)	1 (0.6)
Whorl-double loop	10 (5.6)	4 (2.2)	3 (1.7)	1 (0.6)	2 (1.1)	14 (7.8)	5 (2.8)	2 (1.1)	1 (0.6)	1 (0.6)
Whorl-accidental	1 (0.6)	0 (0.0)	1 (0.6)	0 (0.0)	0 (0.0)	2 (1.1)	1 (0.6)	0 (0.0)	0 (0.0)	0 (0.0)
Arch-plain	9 (5.0)	10 (5.6)	10 (5.6)	6 (3.3)	5 (2.8)	12 (6.7)	14 (7.8)	11 (6.1)	4 (2.2)	9 (5.0)
Arch-tented	0 (0.0)	10 (5.6)	5 (2.8)	1 (0.6)	0 (0.0)	0 (0.0)	16 (8.9)	10 (5.6)	2 (1.1)	0 (0.0)
Total	180 (100)	180 (100)	180 (100)	180 (10)	180 (100)	180 (100)	180 (100)	180 (100)	180 (100)	180 (100)

R, Radical; U, Ulnar.

The significance of the association with impaction was evaluated for all of the study variables. Sex, right-hand middle finger, right-hand ring finger, and left-hand little finger all revealed substantial impaction results. In terms of sex, female subjects and impacted third molars had a 76.7% association (p=0.016). Loop-ulnar and impacted third molar were related with 74.4% in the right-hand middle finger (p=0.024). The whorl-plain and impacted third molar with 60% were related with the right-hand ring finger (p=0.028). Similarly, the loop-ulnar fingerprint was related with both non-impacted and affected third molar teeth with 61.1%; whereas the whorl-plain fingerprint was identified with 33.3% in the impacted third molar tooth group (p=0.009). All of the other study factors, on the other hand, were not linked to impaction ( Table 1).

### Analyses

To predict impaction, a forward stepwise binary logistic regression analysis was used. Impaction was used as a dependent variable, with all versions of fingerprints from all fingers being used as independent variables. All of the fingerprints were grouped into five categories: loop radial, loop-ulnar, whorl-plain, whorl-pocket, whorl-double loop, and arch-plain. The indicator variable was chosen as the first variable-loop radial, and all the others were compared to it. Using impaction as the dependent variable and fingerprints as the predictor variables (categorical), we discovered that the dermatoglyphic pattern of the left-hand little finger pattern was the single most important predictor of impaction, followed by the whorl-plain with an odd ratio of 1 (give the odd ratio with confidence intervals and p-value). Although the whorl-pocket pattern had a high odds ratio, it was not statistically significant. When compared to the loop radial group, whorl-plain had an odds ratio of 6.250 and was more significant for predicting impaction ( Table 4).

**Table 4.** Computation of Odds Ratio.

Variable	B	Sig.	Odds (95% CI for odds)
Left-hand little – Loop-R		0.047	
Left-hand little – Loop-U	1.204	0.079	3.333 (0.870, 12.772)
Left-hand little – Whorl-plain	1.833	0.012	6.250 (1.502, 26.006)
Left-hand little – Whorl-pocket	22.407	1.000	5384916142.837
Left-hand little – Whorl-double loop	-19.999	1.000	0.000
Left-hand little – Arch-plain	-0.875	0.483	0.417 (0.036, 4.813)
Constant	-1.204	0.067	0.300

R, Radical; U, Ulnar.

## Discussion

The purpose to conduct this study was to answer the following clinical question: is there a specific fingerprint pattern linked to impacted third molars? There is a link between dermatoglyphics and mandibular tooth impaction, according to our hypothesis. The aim of this study was to identify the most common dermatoglyphic pattern in people with and without impacted mandibular third molar teeth, as well as any dermatoglyphic markers.

Teeth have a unique histology, making this biomatrix a time capsule for research of prenatal and early life exposure. ^11^ The mandibular third molars are located near the mandible’s posterior and ramus intersection. It is often impacted in the current generation due to various factors and a lack of space. Dermatoglyphics, on the other hand, can be utilized to detect any environmental problems during the foetal stage due to their ectodermal origin. Environmental influences on the symmetry and size of the volar pads, which affects the dermatoglyphic pattern, have been studied in a few studies. ^12^ Dermatoglyphic patterns, like all physical characteristics of the body, are the consequence of a person’s genes and are passed down the generations.

Dermatoglyphics have been linked to a variety of chromosomal abnormalities in a number of studies. In chromosomal illnesses such Patau’s syndrome, Edwards’ syndrome, Down syndrome, and Cri du Chat syndrome, atypical dermatoglyphic patterns can be detected. Dermatoglyphics research in dentistry is still in its early stages, with only a small amount of literature available. Caries, cleft lip and palate, bruxism, malocclusion, periodontal disease, oral submucous fibrosis, and head and neck squamous cell carcinomas are only a few of the dental problems connected to dermatoglyphics. ^2^^,^^13^^,^^14^

Female subjects had higher mandibular third molar impaction than male subjects, according to our findings, and comparable findings were found in a study of the Swedish population. ^15^

When compared to the control group, the whorl-plain pattern in the right-hand ring finger, the loop-ulnar pattern in the right-hand middle finger, and the whorl-plain pattern in the left-hand little finger were found to be significantly linked with the presence of impacted teeth. The single most important predictor for the whorl-plain pattern was the left-hand little finger, which was validated using forward stepwise binary logistic regression. Ramesh *et al.*, discovered a higher frequency of the whorl pattern on the ring and index fingers of the right hand in people with impacted teeth, as well as an increased frequency of the ulnar loop on the index and little finger of the left hand in people who didn’t have any impaction. ^16^ In a similar study investigating for a link between dermatoglyphics and tooth impaction, Narang *et al.*, reported contradicting results, albeit with a small sample size (20 participants). They concluded that in the group with impacted teeth, the tented arch pattern in both hands’ index fingers was more common. ^8^

The non-randomization of the sampling in our study resulted in an unbalanced sex distribution, with female subjects being more likely than male subjects to be in the impacted third molar group. The compliance and patience of the subject during the procedure were the flaws in this investigation. Furthermore, one must be vigilant while recording, as excessive ink use or, on the other side, little ink makes interpreting the pattern harder. The proclivity for finger patterns varies from person to person. Abnormalities in dermal ridges, according to Carter and Matsunaga, can only occur when a combination of inherited and environmental variables exceeds a particular threshold level. ^17^

Various methods to record fingerprints have been discussed in the literature. A few such methods are as follows: i) Ink method (most common method, uses ink and paper); ii) Inkless method (use of a biometric scan machine); iii) Adhesive tape method (uses lead/graphite powder and transparent tape); and iv) Photographic method **(**use of a digital camera). ^18^^–^^20^

The ink approach has been chosen above other existing technologies because of its advantages of being simple, cost-effective, user-friendly, reproducible, safe, and non-invasive. Alternative procedures have their own set of advantages, but the necessary equipment is time-consuming and expensive. Individual fingerprints, including monozygotic twins, are unique and different for each person. Dermatoglyphics can be a reasonably reliable instrument for investigations with a suspected genetic basis. Their benefits, including as ease of use, mass screening, and rapid interpretation, make them a valuable diagnostic tool. From infancy until adulthood, they may be simply recorded. ^18^

When decoding skin carvings, the preference of certain finger patterns to the presence of an impacted tooth may serve as a clinical signal. Impacted molars are more difficult to remove than other diseased teeth, particularly those that are low horizontally and buccally impacted mandibular third molars, which have a high risk of intraoperative and postoperative complications. ^6^ Furthermore, an impacted mandibular third molar reduces the angle of the jaw, making it more prone to fracture, and has been linked to lower arch crowding, temporomandibular joint disorders, vague oro-facial pain, and neuralgias, according to numerous studies. ^21^

In addition, large-scale, multi-racial, multi-geographical population research should be done to reduce variation in a heterogeneous population, as well as to discover the exact process of dermatoglyphic pattern inheritance and its preference for the type of impaction. ^22^ This could help us fine-tune our understanding of differences and allow us to replicate similar studies across human ethnicities. When paired with genetic study, dermatoglyphics research has the potential to uncover more about the genesis and agenesis of third molars. We feel that our findings will give the scientific community a boost, as well as a greater knowledge of how dental anomalies link to dermatoglyphic patterns.

## Conclusions

An impacted tooth can be diagnosed with a clinical examination and a clear radiograph. Dermatoglyphics is used as a preventative approach to forecast the presence of tooth impaction rather than to confirm a diagnosis. The evaluation of the dermatoglyphic pattern is simple, inexpensive, and produces speedy findings. In contrast to symptomatic removal of an impacted tooth, dermatoglyphics could serve as a non-invasive screening method for predicting the existence of impaction, have applications in forensics, and aid in early diagnosis, allowing for more efficient surgical operations with fewer consequences.

Predicting the type of impaction can assist surgeons and patients in understanding the risks of maintaining a distoangular impacted tooth, a horizontally impacted tooth with a high difficulty index and a strong desire to erupt in the usual anatomical position after growth is completed.

Until definitive marker genes for impacted teeth are identified, dermatoglyphic investigation can be combined with other diagnostic techniques and markers to improve diagnosis. DNA testing may be a viable alternative to dermatoglyphics. ^15^

## Data availability

### Underlying data

Figshare: Supplemental Information, https://doi.org/10.6084/m9.figshare.21065809. ^23^

This project contains the following underlying data:

- IMPACTION DATA.xlsx (spreadsheet data)
- English information.pdf (information sheet)

Data are available under the terms of the Creative Commons Zero “No rights reserved” data waiver (CC0 1.0 Public domain dedication).
