# Supplementary material for: Evaluation of the relationship between dermatoglyphics and mandibular third molar impaction: A cross-sectional study
Source: F1000Res. 2022 Sep 29;11:1120. [Version 1] doi: 10.12688/f1000research.123398.1 (PMC10465997; doi:10.12688/f1000research.123398.1)
Supplement: Peer review history

# Peer review: Reviewer response for version 1

**DOI:** 10.5256/f1000research.135500.r178068

**Published:** 2023-08-29

**Authors:** Kalyani Bhate

**Affiliations:** 1 Department of Oral and Maxillofacial Surgery, Dr. D. Y. Patil Dental College and Hospital, Dr. D. Y. Patil Vidyapeeth, Pune, Maharashtra, India

The article by Kapoor *et al.* discusses common dermatoglyphic patterns to predict the presence of impacted teeth. Different patterns on different fingers are associated with a predictive value. The article appears to positively co-relate certain fingerprint patterns on certain fingers to the presence of impacted teeth. This is statistically significant in their paper and this unique finding needs to be corroborated with larger sample sizes. While it may not aid clinicians clinically in managing impacted teeth, the theoretical treatise seems unique and adds value to the literature. It appears to be an unexplored venue and needs further exploring to ascertain whether it is a coincidence.

**Major Comments:**

 Statistical analysis seems robust. The findings seem to be backed by a sound statistical methodology. References are relevant and buttress the article. A simple ink approach was used that appears to be effective. Dermatoglyphics are certainly more cost effective than DNA testing for prediction. However, how this prediction may help has to come out more clearly in the paper.

**Minor Comments:**

 The authors could expand on how this knowledge and findings could translate to benefit patients and clinicians.

 The article is well drafted and follows a clear flow of thought. The language is lucid. The article is a theoretical treatise but it seems to be done following due protocol and an appropriate analysis.

 While it may not have huge impact on translation, it serves to facilitate discussion on an interesting aspect of the relation of finger prints to impacted third molars. The area needs to be further explored with RCTs and it is this purpose that the article serves.

Is the work clearly and accurately presented and does it cite the current literature?

Yes

If applicable, is the statistical analysis and its interpretation appropriate?

Yes

Are all the source data underlying the results available to ensure full reproducibility?

Yes

Is the study design appropriate and is the work technically sound?

Yes

Are the conclusions drawn adequately supported by the results?

Yes

Are sufficient details of methods and analysis provided to allow replication by others?

Yes

Reviewer Expertise:

Impacted Teeth, minor oral surgery, anthropology

I confirm that I have read this submission and believe that I have an appropriate level of expertise to confirm that it is of an acceptable scientific standard.

# Peer review: Reviewer response for version 1

**DOI:** 10.5256/f1000research.135500.r187581

**Published:** 2023-08-23

**Authors:** Nisha Ashifa

**Affiliations:** 1 Annamalai University, Annamalai Nagar, Tamil Nadu, India

I appreciate the authors for presenting a well-conducted and well-written study. The article explains dermatoglyphics and their uses/associations in detail. The methodology is clearly interpreted which allows for reproducibility. Statistical analysis seems to be appropriate. The discussion and conclusion are adequate. There are a few suggestions/remarks I would like to add, to improve the current article. 

1. Can the possibility of future impactions be diagnosed before the eruption of wisdom teeth since the fingerprints are developed at birth and remain stable throughout the life span? 
2. Photos of each type of fingerprint pattern could've been added to the article, enabling easy visualization and interpretation for the readers. 
3. Can you please explain why such study populations have been included in the study? Have you included patients with clinically and radiographically missing third molars in the study group? I feel that area needs clarification. 
4. Was any criteria used to diagnose Impactions? Like WAR lines or Winters classification?
5. Does the results of the study give insight to the surgeons in predicting the difficulty of impaction?

Is the work clearly and accurately presented and does it cite the current literature?

Yes

If applicable, is the statistical analysis and its interpretation appropriate?

I cannot comment. A qualified statistician is required.

Are all the source data underlying the results available to ensure full reproducibility?

Yes

Is the study design appropriate and is the work technically sound?

Yes

Are the conclusions drawn adequately supported by the results?

Yes

Are sufficient details of methods and analysis provided to allow replication by others?

Partly

Reviewer Expertise:

Periodontology

I confirm that I have read this submission and believe that I have an appropriate level of expertise to confirm that it is of an acceptable scientific standard.
